# Impact of retrospective data verification on the results of the academic-led ICON6 trial

**DOI:** 10.1186/1745-6215-16-S2-P184

**Published:** 2015-11-16

**Authors:** Andrew Embleton, Elizabeth Clark, Stephen Townsend, Laura Farrelly, Cheryl Jones, Richard Kaplan

**Affiliations:** 1 MRC Clinical Trials Unit at UCL, London, UK; 2 CRUK and UCL Cancer Trials Centre, London, UK

## 

ICON6 (ISRCTN68510403) is a phase III academic-led international double-blind placebo-controlled randomised trial of the addition of cediranib to chemotherapy in recurrent ovarian cancer. The trial established a beneficial gain in progression-free survival (PFS), conducted using the long established risk-based monitoring model as advocated by FDA/EMA as an alternative to the traditional monitoring-intensive industry approach. AstraZeneca are currently considering regulatory submissions using ICON6 as the single pivotal trial.

Given the lower level of on-site monitoring performed AstraZeneca mandated the CTU implement retrospective Source Data Verification (SDV) of medical records against Case Report Forms (CRFs), and complete Quality Control (QC) checks of single data entry. Additionally, Blinded Independent Central Review (BICR) of imaging studies to assess investigator ascertainment bias was performed. We summarise changes resulting from additional monitoring and the impact on reported results.

Amongst 253 events in 282 patients in the primary comparison two additional progressions were reported, one in each arm. The result of the log-rank test changed marginally, remaining p<0.001. No change in a hazard ratio of 0.57, with minor confidence interval adjustment from 0.45-0.74 to 0.44-0.73. Reference median time-to-event remained at 8.7 months, with the comparator revised from 11.1 to 11.0. Within those progressions assessed radiographically, BICR identified differences as expected given the subjective nature of scan assessment. However these differences were slight.

SDV, QC and BICR were resource intensive and an additional burden to both site and CTU staff and, as anticipated, resulted in immaterial data changes with a minimal gain in accuracy of key study endpoints.

